# Investigating efficacy of colchicine plus phenolic monoterpenes fraction as a potential treatment for patients diagnosed with COVID-19: A randomized controlled parallel clinical trial

**DOI:** 10.1016/j.heliyon.2024.e27373

**Published:** 2024-03-06

**Authors:** Siavash Vaziri, Alireza Janbakhsh, Mohammad Hossein Zamanian, Yadollah Shakiba, Shayan Mostafaei, Amir Hossein Norooznezhad, Kamran Mansouri, Ahmad Bagheri, Farhad Abdali, Kavyan Fatahpour, Ali Mostafaie

**Affiliations:** a Department of Infectious Diseases, School of Medicine, Kermanshah University of Medical Sciences, Kermanshah, Iran; b Medical Biology Research Center, Health Technology Institute, Kermanshah University of Medical Sciences, Kermanshah, Iran; c Department of Biostatistics, School of Health, Kermanshah University of Medical Sciences, Kermanshah, Iran; d Epidemiology and Biostatistics Unit, Rheumatology Research Center, Tehran University of Medical Sciences, Tehran, Iran

**Keywords:** COVID-19, Colchicine, Phenolic monoterpenes, Complementary treatment

## Abstract

**Background:**

COVID-19 now is a serious concern for the world healthcare system. This study aimed to investigate possible therapeutic effect of colchicine and phenolic monoterpenes accompanied by standard care of treatment (SCT) in patients diagnosed with COVID-19.

**Methods:**

In this randomized controlled parallel clinical trial, a total number of 179 (of 200) patients with confirmed COVID-19 were enrolled according to the inclusion and exclusion criteria. The patients were allocated by simple randomization method into two groups control (receiving SCT with 71 patients) and intervention (receiving SCT plus colchicine and phenolic monoterpenes with 107 patients). The mortality ratio during hospitalization as well as a 2-week follow-up, ICU admission rate, and hospitalization duration were assessed as main outcomes.

**Results:**

The mortality ratio was 0.9% (1/108) and 8.45% (6/71) in the intervention and the control groups (p-value = 0.035) respectively, these ratios after a 14-day follow-up were 1.85% (2/108), and 9.85 (7/71) respectively (p-value = 0.031). Also, the ICU admission was significantly lower (p-value = 0.006) in the intervention group 2/108 (1.85%) compared with controls 10/71 (14.08%). Moreover, the duration of hospitalization followed a similar pattern to ICU admission with 4.17 ± 1.34 vs. 6.39 ± 2.59 days in the intervention and control groups respectively (p-value< 0.001). Furthermore, no significant side effect was found between the groups.

**Conclusion:**

According to the results, the combination of colchicine plus phenolic monoterpenes could be an additive treatment for the SCT. The authors strongly recommend further trials on this combination with other SCTs.

## 1 Introduction

Coronavirus disease 2019 (COVID-19) which started in China in late 2019, led to a pandemic emergency according to the declaration of the World Health Organization (WHO). This disease has infected millions of people and caused many morbidities and even mortality. COVID-19 which is caused by a virus named severe acute respiratory syndrome coronavirus 2 (SARS-CoV-2) may cause different signs and symptoms in different age groups [1]. The main clinical manifestation of COVID-19 has been known to be pneumonia [2], however, this disease could cause different morbidities such as acute respiratory distress syndrome (ARDS), septic shock, acute kidney injury, acute cardiac injury, heart failure, and hepatic failure [3]. Despite the clinical manifestation, different findings such as changes in leukocyte count, lymphopenia, decrease in absolute neutrophil count, and elevated levels of C reactive protein (CRP) have been seen in the primary laboratory investigation of patients diagnosed with COVID-19 [4]. Other than clinical trials performed on available medications, different trials have been performed on natural-derived products on patients with COVID-19 [5]

This randomized controlled parallel clinical trial aimed to investigate possible efficacy of colchicine plus phenolic monoterpenes fractions as a potential side treatment for patients diagnosed with COVID-19.

## 2 Methods and patients

### 2.1 Study design

**Fig. 1:**
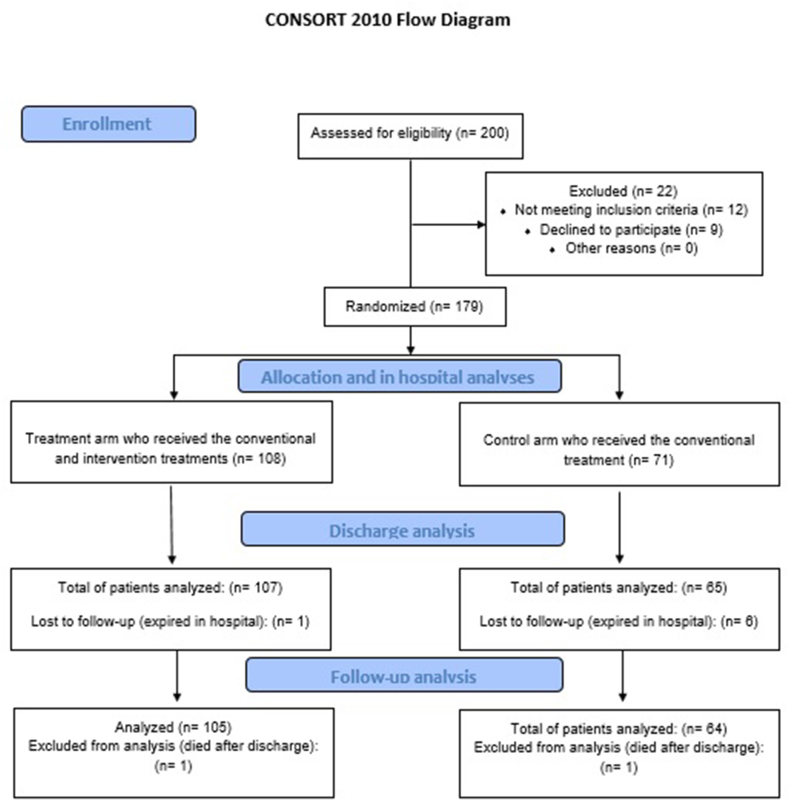
Study design and protocol.

This randomized controlled parallel clinical trial was performed in two COVID-19 special hospitals in Kermanshah, Iran from April 2020 to December 2020. Inpatients individuals older than 10 years old with a confirmed diagnosis of COVID-19 according to the SARS-CoV-2 real-time polymerase chain reaction (RT-PCR) and/or lung computed tomography (CT) scan were evaluated for further assessments. The exclusion criteria were defined as pregnancy and severe chronic liver disease. Patients who met both inclusion and exclusion criteria enrolled in the study and allocated in a ratio of 1.5 to 1 (intervention to control) through simple randomization method (Fig. 1).

### 2.2 Ethics

The aims and methods of this study were clearly explained to patients or their legal guardians (in case of being less than 18 years old) according to their level of knowledge and education and they were asked to sign a consent form freely following the explanations. This study was approved by the Medical Ethics Committee of Kermanshah University of Medical Sciences. Also, it has been recorded in the Iranian Registry of Clinical Trial (IRCT20150623022884N3) and clinicaltrials.gov (NCT04392141). All the authors adhered to the Helsinki 1964 Declaration and its further revisions.

### 2.3 Definitions

Fever was defined according to the guidelines by the Centers for Disease Control and Prevention (CDC) [6] and the Iranian Ministry of Health and Medical Education [7] as body temperature of >38 °C and >37.8 °C respectively. The severe COVID-19 was defined according to the National Institute of Health (NIH) definition [8].

### 2.4 Intervention materials

The control group received standard care of treatment (SCT) according to the university guideline which was lopinavir/ritonavir (Kaletra®). The intervention groups received a combination of colchicine plus phenolic monoterpenes. The phenolic monoterpenes were extracted from *Nigella sativa* and *Trachyspermum ammi* based on a total phenolic standard. Also, colchicine was extracted from *Colchicum autumnale*. The intervention group received a total of 0.8 mg/day of colchicine as well as 45 mg of the mentioned extracted phenolic monoterpenes plus Kaletra®. The duration of the treatment of colchicine plus phenolic monoterpenes was 12 days. The intervention treatments were provided by Zist Tolid Razi Co (Kermanshah, Iran).

### 2.5 Data collection

The demo-biographic data (sex and age), as well as any underlying condition, were collected from the patients’ files. Patients in each group were evaluated for COVID-19-related signs and symptoms on the admission day. Also, complete blood count (CBC) variables, C reactive protein (qualitative), and lactate dehydrogenase (LDH) were investigated among the routine laboratory evaluations. Hospitalization duration, intensive care unit (ICU) admission, and mortality ratio were considered as the main outcomes. Moreover, changes in the signs including body temperature and SpO_2_ evaluated in both groups on the third day of receiving treatment and discharge/death day (compared to their admission time as the baseline).

### 2.6 Statistical analysis

All the data were collected in Microsoft Excel (Microsoft®) and controlled by two investigators separately. The continuous and categorical variables were presented as mean ± standard deviation (SD) and N (%) respectively. After checking the normality assumption, independent-sample T-test and paired T-test were used for comparing the continuous variables between groups and before and after analyses, respectively. Also, Chi-square/Fisher exact test was applied to assess the associations of the categorical variables and both groups. Data analyses were performed by SPSS version 25 (SPSS Inc, Chicago, IL, USA). The significance level was set at the level of 0.05.

## 3 Results

### 3.1 Baseline characteristic

According to both inclusion and exclusion criteria from a total number of 200 patients, 179 individuals were included in the trial. For the SCT and intervention groups, 71 and 108 patients were allocated, respectively. The control group's mean age was 54.21 ± 15.39 which had no significant difference (p-values = 0.527) compared to the intervention group (52.71 ± 15.52). Also, the ratio of females in the control and intervention groups were 37 (52.1%) and 55 (50.9%) respectively which showed no statistical differences for gender (p-values = 0.876) among the two arms. In control and intervention groups, 36 (50.7%) and 62 (57.4%) (respectively) of patients had underlying diseases such as hypertension, diabetic mellitus, asthma, and malignancy which revealed no statistically significant difference (p-value = 0.378) between the groups. The details of underlying diseases/disorders have been provided in Table 1. Also, primary evaluations at admission of the patients showed no statistically significant difference in variables including leukocyte and lymphocyte number, platelet count, hemoglobin (Hb) and LDH levels, and body temperature (all p-values> 0.05) between the groups. However, regarding the SpO_2_, the control group (87.39 ± 6.32) has a significantly lower mean (p-values< 0.001) compared to the intervention group (90.94 ± 3.27) at the admission phase. Considering this finding, the severity of the disease was investigated between the groups according to the NIH guideline [8], which showed that 62 (88.57%) and 90 (83.33%) individuals in the control and the intervention groups had a severe illness which revealed no significant difference (p-values = 0.510) between the groups.

**Table 1:** Demographic and underlying diseases in the control and SCT intervention groups.

Variable	Control (n = 71)	Intervention (n = 108)	P-value
Age (years)	54.21 ± 15.39	52.71 ± 15.52	0.527
Female	37 (52.1%)	55 (50.9%)	0.876
Male	34 (47.9%)	53 (49.1%)	
Underlying disease (overall)	36 (50.7%)	62 (57.4%)	0.378
Hypertension	12 (16.9%)	26 (24.0%)	0.251
Other cardiovascular diseases	5 (7.0%)	11 (10.2%)	0.596
Diabetic mellitus	5 (7.0%)	11 (10.2%)	0.596
Asthma	1 (1.4%)	2 (1.8%)	>0.999
Malignancy	2 (2.8%)	7 (6.4%)	0.322
End stage renal disease/CKD	3 (4.2%)	9 (8.3%)	0.368
Immunosuppressive diseases	0	1 (0.9%)	>0.999
Autoimmune diseases	4 (5.6%)	10 (9.2%)	0.571
Chronic liver diseases	1 (1.4%)	5 (4.6%)	0.405
Hypothyroidism	0	1 (0.9%)	>0.999
Hyperthyroidism	1 (1.4%)	0	0.397
Anemia	0	2 (1.8%)	0.519
Recent pneumonia	4 (5.6%)	8 (7.4%)	0.766
Smoking/Substance abuse	2 (2.8%)	12 (11.1%)	0.049
Sever to critically ill COVID-19	62/71 (87.3%)	90/107 (84.1%)	0.510

### 3.2 Clinical outcomes

As mentioned, different clinical variables in both groups were studied during the hospitalization period. The mortality ratio in this duration in the intervention group (1/108 [0.9%]) represented a significantly lower ratio (p-values = 0.035) compared to the control group (6/71 [8.45%]). Moreover, the accumulative mortality ratio after 14 days follow-up following discharge was significantly lower (p-values = 0.031) in the intervention group (2/108 [1.85%]) compared to the control arm (7/71 [9.85%]). Also, in the intervention group, a significant decrease in ICU admission was observed compared to the control group (2/108 [1.85%] vs. 10/71 [14.08%] respectively, p-values = 0.006). Furthermore, the intervention group (4.17 ± 1.34 days) showed to has a significantly (p-value< 0.001) lower hospitalization duration than the control arm (6.93 ± 2.59 days). Regarding the other clinical variables, statistical analyses showed a narrow p-value of 0.051 for the difference in SpO_2_ levels in the intervention (93.49 ± 2.12%) and the control groups (92.98 ± 1.76%) at the discharge moment. The details of clinical outcomes have been provided in Table 2. According to Supplementary Tab. 1, there was no serious side effect in the intervention group compared to the control arm (p values > 0.05).

**Table 2:** Clinical outcomes of the studied groups.

Parameter		Intervention (n = 108)	Control (n = 71)	Odds ratio (95% CI)	P-value
Mortality rate (during hospitalization)		1 (0.9%)	6 (8.45%)	9.87 (1.16–83.89)	**0.035**
Mortality rate (After 14 days follow-up)		2 (1.85%)	7 (9.85%)	5.79 (1.16–28.76)	**0.031**
ICU admission rate		2 (1.85%)	10 (14.08%)	8.68 (1.84–40.95)	**0.006**
Hospitalization Duration (Days)		4.17 ± 1.34	6.39 ± 2.59	Not applicable	**<0.001**
SpO_2_ (%)	Admission	90.94 ± 3.27	87.39 ± 6.32	Not applicable	**<0.001**
	Discharge	93.49 ± 2.12***	92.89 ± 1.76***	Not applicable	0.051
Body temperature (°C)	Admission	37.74 ± 0.43	37.67 ± 0.40	Not applicable	0.695
	Discharge	37.37 ± 0.38***	37.0 ± 0.41***	Not applicable	**0.004**

95% CI: 95% confidence interval. ***: P-value<0.001 for before and after evaluation of each variable.

### 3.3 Paraclinical outcomes

As mentioned, different laboratory variables were evaluated in the studied groups. Discharge absolute lymphocyte count in the intervention group (1.74 ± 0.83 cells × 10^3^/mm^3^) was significantly (p value < 0.001) higher than the control group (1.17 ± 0.49 cells × 10^3^/mm^3^). Also, similar results were observed for the discharge lymphocyte to leukocytes ratio (27.0 ± 9.38 vs 18.66 ± 8.55, p value < 0.001). Moreover, LDH levels at discharge were significantly lower (p-value = 0.004) in the intervention group (542.94 ± 225.67 U/L) compared to the SCT group (691.04 ± 299.09 U/L). Furthermore, the statistically significant increase in the mean of absolute lymphocyte count (p value < 0.001) and lymphocyte to leukocytes ratio (p value < 0.001) as well as a decrease in the LDH levels (p value < 0.001) compared to their baseline levels were only observed in the intervention group (details have been provided in Table 3). The significance of differences between admission and discharge values of laboratory variables in each group have been shown in Table 3).

**Table 3:** Comparisons of laboratory tests between the studied groups.

Parameter	Time	Intervention (n = 108)	Control (n = 71)	P-value
Leukocyte × 10^3^/mm^3^	Admission	7.08 ± 3.65	7.11 ± 3.57	0.954
	Discharge	6.87 ± 3.57	7.35 ± 3.70	0.415
Lymphocytes/leukocyte (%)	Admission	20.91 ± 8.82	19.55 ± 7.98	0.323
	Discharge	27.0 ± 9.38***	18.66 ± 8.55	**<0.001**
Absolute lymphocytes × 10^3^/mm^3^	Admission	1.31 ± 0.64	1.19 ± 0.45	0.175
	Discharge	1.74 ± 0.83***	1.17 ± 0.49	**<0.001**
Platelet × 10^3^/mm^3^	Admission	199.82 ± 115.62	200.26 ± 104.34	0.980
	Discharge	224.08 ± 112.42*	222.80 ± 86.15***	0.939
Hemoglobin (gr/dL)	Admission	12.0 ± 2.21	12.55 ± 1.92	0.594
	Discharge	11.43 ± 2.05***	11.59 ± 1.71***	0.159
LDH (U/L)	Admission	680.27 ± 471.44	664.98 ± 295.84	0.836
	Discharge	542.94 ± 225.67***	691.04 ± 299.09	**0.004**

*P-value<0.05, **P-value<0.01, and ***P-value<0.001 represent p-values for difference changes between before/admission and after/discharge analysis for each variable of a group.

## 4 Discussion

This randomized controlled clinical trial showed that SCT plus colchicine and phenolic monoterpene-rich fractions seem to be more effective just than SCT. The intervention groups had a lower mortality ratio in both hospitalization duration and 2-week follow-up. Also, the admission to ICU was lower in the intervention group compared to the controls. Moreover, the intervention group showed important significant improvement in different assessed laboratory variables.

As mentioned, both groups had no statistically significant difference in age, sex, and underlying diseases (overall and one by one). The only difference observed was in the SpO_2_ levels which we have decided to evaluate the disease severity according to the NIH criteria [8] that showed no statistically significant difference among the groups regarding the frequency of patients with severe state of disease. As the results showed, the mortality ratio during hospitalization in the control group was 8.45% which was very similar to the obtained results of 431 hospitalized patients with COVID-19 (9%) reported in the same city and same condition [1]. Thus, it seems that despite the lower number of controls compared to the intervention group, the controls were the well-represented target population.

A randomized controlled trial on the efficacy of colchicine in patients diagnosed with COVID-19 in two groups of intervention (55 cases) vs. standard care of treatment (50 cases) showed significant improvement in time to clinical deterioration. They found event-free 10-day survival for control and intervention groups were 83% vs. 97% (p-value = 0.03) respectively [9]. Moreover, another randomized, double-blinded, placebo-controlled clinical trial has been performed on colchicine and COVID-19 in two 36 cases arms. It has been shown that colchicine was able to significantly reduce the need for supplemental oxygen and length of hospitalization in the intervention group compared to the controls [10]. Also, another randomized control clinical trial is now conducting on the potential of *Nigella sativa* supplementation to treat symptomatic mild COVID-19 but no result has been published for it. It has been stated that *Nigella sativa* oil (MARNYS® Cuminmar) softgel in dosage of 500 mg each 12-h (BID) for ten days [11].

Colchicine is a well-known anti-inflammatory agent that has been used in medicine for a long time. In the therapeutic dose, colchicine could decrease crucial steps of inflammation such as migration, degranulation, and cell-cell attachment in the inflammatory-related cells especially macrophages and neutrophils [12]. The phenolic monoterpenes such as thymoquinone have shown strong antioxidant and anti-inflammatory properties [13]. Using the phenolic monoterpenes along with colchicine not only synchronously increases the anti-inflammatory action of colchicine but also might decrease any possible cytotoxic effect. Also, the anti-oxidant role of phenolic monoterpenes should not be ignored [13] since oxidative stress is a key role player in the many aspects of COVID-19 pathogenesis [14] especially endothelial dysfunction that leads to thromboembolic events [15].

As same as any other study, this one has its limitations. The main limitation of this study was the low sample size of the studied patients. Also, the current study used colchicine plus phenolic monoterpenes fraction which the exact compounds of phenolic monoterpenes fraction are not well-known. Moreover, the current investigation was an open-label clinical trial which could be another limitation.

Although the results of the study were suggestive to using the mixture of colchicine accompanied by phenolic monoterpenes as a possible treatment for COVID-19, it seems further studies such as double-blinded randomized control trials on both inpatients and outpatients are necessary. One of the missing points of this study was the lack of some laboratory data such as interleukin 6 (IL-6), quantitative C-reactive protein, and cardiac troponin values which should be evaluated in further studies.

## 5 Conclusion

According to the results, the combination of colchicine and phenolic monoterpenes could be a possible side treatment for COVID-19. This treatment option showed decrease in mortality ratio, ICU admission rate, and hospitalization duration in the treated patients. The authors of this study suggest further investigations on this mixture as a potential co-treatment for COVID-19.

## Funding

This study was funded by Zist Tolid Razi Co (Kermanshah, Iran).

## CRediT authorship contribution statement

**Siavash Vaziri:** Writing – review & editing, Writing – original draft, Project administration, Investigation, Data curation. **Alireza Janbakhsh:** Writing – review & editing, Writing – original draft, Investigation. **Mohammad Hossein Zamanian:** Writing – review & editing, Writing – original draft, Investigation. **Yadollah Shakiba:** Writing – review & editing, Writing – original draft, Investigation. **Shayan Mostafaei:** Writing – review & editing, Writing – original draft, Visualization, Software, Methodology. **Amir Hossein Norooznezhad:** Writing – review & editing, Writing – original draft, Methodology, Investigation. **Kamran Mansouri:** Writing – review & editing, Writing – original draft, Investigation. **Ahmad Bagheri:** Writing – review & editing, Writing – original draft, Investigation. **Farhad Abdali:** Writing – review & editing, Writing – original draft, Investigation. **Kavyan Fatahpour:** Writing – review & editing, Writing – original draft, Investigation. **Ali Mostafaie:** Writing – review & editing, Writing – original draft, Project administration, Investigation, Funding acquisition, Data curation, Conceptualization.

## Declaration of competing interest

The authors declare that they have no known competing financial interests or personal relationships that could have appeared to influence the work reported in this paper.
