# Supplementary material for: Investigating efficacy of colchicine plus phenolic monoterpenes fraction as a potential treatment for patients diagnosed with COVID-19: A randomized controlled parallel clinical trial
Source: Heliyon. 2024 Mar 6;10(6):e27373. doi: 10.1016/j.heliyon.2024.e27373 (PMC10955262; doi:10.1016/j.heliyon.2024.e27373)
Supplement: Multimedia component 1 [file mmc1.docx]

**Supplementary table**

**Table 1.** Side effect of the treatments in both groups.

| **Side effect** | **Standard care of treatment (SCT)** | **SCT and additive therapy** | **P-value** |
| --- | --- | --- | --- |
| Nausea/vomiting | 3/71 (4.22%) | 4/107 (3.73%) | >0.999 |
| Restlessness | 2/71 (2.81%) | 2/107 (1.86%) | >0.999 |
| Constipation | 1/71 (1.40%) | 2/107 (1.86) | >0.999 |
